# Inbreeding, inbreeding depression, and infidelity in a cooperatively breeding bird*

**DOI:** 10.1111/evo.13496

**Published:** 2018-07-05

**Authors:** Gabriela K. Hajduk, Andrew Cockburn, Nicolas Margraf, Helen L. Osmond, Craig A. Walling, Loeske E. B. Kruuk

**Affiliations:** ^1^ Institute of Evolutionary Biology, School of Biological Sciences University of Edinburgh Edinburgh United Kingdom; ^2^ Division of Evolution and Ecology, Research School of Biology The Australian National University Canberra ACT Australia; ^3^ Current Address: Nicolas Margraf, Musée d'histoire naturelle de La Chaux‐de‐Fonds Av. Léopold‐Robert 63 CH‐2300 La Chaux‐de‐Fonds Switzerland

**Keywords:** Cooperative breeding, extra‐pair, inbreeding depression, inbreeding avoidance, infidelity

## Abstract

Inbreeding depression plays a major role in shaping mating systems: in particular, inbreeding avoidance is often proposed as a mechanism explaining extra‐pair reproduction in socially monogamous species. This suggestion relies on assumptions that are rarely comprehensively tested: that inbreeding depression is present, that higher kinship between social partners increases infidelity, and that infidelity reduces the frequency of inbreeding. Here, we test these assumptions using 26 years of data for a cooperatively breeding, socially monogamous bird with high female infidelity, the superb fairy‐wren (*Malurus cyaneus*). Although inbred individuals were rare (∼6% of offspring), we found evidence of inbreeding depression in nestling mass (but not in fledgling survival). Mother–son social pairings resulted in 100% infidelity, but kinship between a social pair did not otherwise predict female infidelity. Nevertheless, extra‐pair offspring were less likely to be inbred than within‐pair offspring. Finally, the social environment (the number of helpers in a group) did not affect offspring inbreeding coefficients or inbreeding depression levels. In conclusion, despite some agreement with the assumptions that are necessary for inbreeding avoidance to drive infidelity, the apparent scarcity of inbreeding events and the observed levels of inbreeding depression seem insufficient to explain the ubiquitous infidelity in this system, beyond the mother–son mating avoidance.

It is often expected that inbreeding depression should generate selection to avoid inbreeding (Pusey 1987; Blouin and Blouin 1988; Tregenza and Wedell 2000; Szulkin et al. 2013). In particular, inbreeding avoidance has frequently been suggested as an explanation for female infidelity in socially monogamous species. If individuals are likely to be socially paired with a relative, extra‐pair paternity (EPP) may have adaptive advantages if it reduces rates of inbreeding (Blomqvist et al. 2002; Foerster et al. 2003). The expectation that inbreeding depression is ubiquitous in diploids (Lynch and Walsh 1998) and the difficulties inherent in explaining the occurrence of EPP (Griffith et al. 2002; Westneat and Stewart 2003; Arnqvist and Kirkpatrick 2005; Forstmeier et al. 2014) have jointly strengthened the appeal of this “inbreeding‐avoidance hypothesis” for the occurrence of extra‐pair reproduction. However, quantifying the relevant parameters in empirical studies is challenging, and so our understanding of several key aspects of the interplay between inbreeding and infidelity in natural populations is still limited. Here, we use data from a long‐term study to investigate the effects of exceptionally high rates of extra‐pair paternity on inbreeding and inbreeding depression in a passerine bird.

Three assumptions are necessary to support the notion that extra‐pair mating occurs to facilitate inbreeding avoidance: (1) that inbreeding depression is present; (2) that infidelity increases with kinship to social mate; and (3) that infidelity reduces the chances of inbreeding. We emphasize that while these assumptions are necessary for there to be adaptive benefits of inbreeding avoidance *via* EPP (i.e., these will not occur without the assumptions being met), they may not be sufficient (i.e., the assumptions being met does not inevitably guarantee the outcome). In particular, with regard to (1), the existence of inbreeding depression may not inevitably select for inbreeding avoidance because of the potential inclusive fitness benefits of inbreeding (through increased reproductive success of relatives; e.g., Bengtsson 1978; Parker 1979; Kokko and Ots 2006; Duthie and Reid 2015, 2016). Increased, rather than decreased, rates of inbreeding *via* extra‐pair paternity may even be adaptive if inbreeding depression is sufficiently mild (Lehtonen and Kokko 2015). Further, the overall benefits of inbreeding avoidance will also depend on the potential costs of any avoidance mechanisms (Koenig et al. 1999; Lehmann and Perrin 2003). The selection pressures on the alternative mating strategies of inbreeding avoidance, inbreeding preference or random mating will therefore depend on the relative magnitudes of inbreeding depression, the benefits to inclusive fitness of inbreeding, and the costs of inbreeding avoidance (Szulkin et al. 2013; Duthie and Reid 2016; Duthie et al. 2016a). However, in setting out a necessary (if not sufficient) set of conditions, assumptions (1)—(3) provide a useful framework for evaluating the plausibility of inbreeding avoidance *via* extra‐pair paternity.

Studies of wild populations will be especially valuable for evaluation of mating patterns and inbreeding, because laboratory studies may not be able to recreate natural patterns of mate choice, and also because inbreeding depression may change with environmental conditions (Crnokrak and Roff 1999; Joron and Brakefield 2003; Szulkin and Sheldon 2007). Studies to date have provided evidence from wild populations for each of the three assumptions outlined above. For example, Keller and Waller (2002) review evidence for inbreeding depression in the wild (assumption 1); Leclaire et al. (2013) and Arct et al. (2015) review evidence across species for relatedness to the social mate increasing EPP (assumption 2); and Foerster et al. (2003) and Reid et al. (2015b) document reduced inbreeding coefficients as a result of infidelity in two passerine bird species. However, comprehensive tests of all three assumptions within a single study system are scarce. One notable exception is the work on a Canadian population of song sparrows (*Melospiza melodia*), where evidence combined across several papers covers all three assumptions (assumption 1: e.g., Taylor et al. 2010; assumptions 2 and 3: Reid et al. 2015a,b,c). However, the song sparrow study involves a small, isolated island population where high levels of relatedness between individuals are expected and observed. Equivalent studies of systems with other characteristics will therefore be required for any indication of the generality of these patterns.

The scarcity of comprehensive empirical studies of the role of extra‐pair (EP) reproduction in facilitating inbreeding avoidance may be partially due to the inherent difficulty of quantifying inbreeding and inbreeding depression in the wild. Analyses of inbreeding require estimates of relatedness between individuals, but in socially monogamous systems, social pedigrees based on the observed parental behavior cannot provide accurate estimates of relatedness when infidelity is present, meaning that genetic information of some form is ideally required. Several studies of associations between extra‐pair mating and relatedness (assumption 2) have used genetic rather than social data, but these are typically based on assessing inbreeding from heterozygosity at a handful of molecular markers, typically microsatellites (e.g., Smith et al. 2005; Foerster et al. 2006). This is potentially problematic as heterozygosity at a small number of markers may be only weakly correlated with genome‐wide heterozygosity: just as it is now clear that inbreeding depression cannot reliably be estimated from correlations between heterozygosity of a few markers and trait values (Balloux et al. 2004; Slate et al. 2004; Szulkin et al. 2010), studies that use a low number of markers to test for inbreeding avoidance through EP matings may not be able to estimate relevant levels of relatedness sufficiently accurately. Furthermore, estimates of inbreeding and relatedness may be marker dependent (Wang 2014), and using the same markers to evaluate both paternity and heterozygosity may lead to false positives when assessing the role of heterozygosity in mate choice (Wetzel and Westneat 2009). These issues mean that genetically informed pedigrees and/or high‐density genomic data are required for accurate estimates of relatedness, inbreeding, and inbreeding depression (Pemberton 2004; Harrison et al. 2013), and hence, for accurate tests of the hypothesis of inbreeding avoidance through EP reproduction in the wild.

The dynamics of inbreeding, inbreeding depression, and infidelity may also be shaped by the social environment of individuals, in particular the number and characteristics of conspecifics with which they interact (Koenig and Haydock 2004). In cooperatively breeding species that live in groups of closely related individuals, group composition may affect mating patterns and change any effects of inbreeding depression on offspring development. The social environment may affect the chances that an individual inbreeds: the likelihood that relatives will be socially paired may be higher in cooperative breeders where close adult kin are tolerated in the social group, than in other social systems. The hypothesis of inbreeding avoidance through EP matings assumes that extra‐pair partners will be less‐closely related than social partners. However, this may not be so if the closest available alternative mate is equally related––a scenario that can readily occur in cooperatively breeding groups. Thus, immediate social environment may play an important role in shaping inbreeding and infidelity patterns.

There is also a general expectation that inbreeding depression may vary with environmental conditions (e.g., Miller 1994; Armbruster and Reed 2005; Fox and Reed 2010), although detecting inbreeding‐environment interactions has been difficult in wild populations (Pemberton et al. 2017). We might thus also expect inbreeding depression to vary with social environment. Recent theoretical work suggests parents should invest more care into inbred offspring to counteract the reduced viability of such offspring (Duthie et al. 2016b)––by extension, if assisted parents in cooperatively breeding species can rely on helpers to provide additional care for offspring, potentially lessening the effects of inbreeding depression, selection against inbreeding could be reduced. Whether this occurs in natural populations is not yet clear: for example, a recent study of inbreeding in meerkats (Nielsen et al. 2012) found positive effects of helpers but negative effects of inbreeding depression on offspring growth, but did not find evidence that helpers mitigated the negative effects of inbreeding depression. However, such associations may play an important role in the impact of social environment on inbreeding‐infidelity dynamics.

In this article, we investigate the intraspecific relationship between inbreeding and infidelity, and its interaction with the social environment. Our study species, the superb fairy‐wren (*Malurus cyaneus)*, is socially monogamous but has exceptionally high levels of female infidelity, effectively making it the least faithful species of the least faithful bird genus (Cockburn et al. 2016). We used data from a long‐term study of a wild population in south‐east Australia, including a genetically based pedigree to estimate levels of inbreeding and inbreeding depression.

Superb fairy‐wrens are characterized by substantial variation in levels of cooperative breeding. About half (54.5%) of breeding attempts in the population involve just a single pair of breeding adults, whereas the other half are helped by up to four (exceptionally rarely five) male subordinates (or “helpers”), frequently sons from previous breeding attempts (61.8% of all helpers are sons of the breeding female). This cooperative breeding increases the chances of social pairing between relatives: helper males form a stable queue, and may inherit the dominant position and thus pair socially with their mother (Cockburn et al. 2008b). Individual females will therefore experience different social environments, dependent on whether they are breeding just as a pair or are accompanied by helpers, and whether they are socially paired to their son or not.

The current study is motivated by the observation that mothers never produce offspring with their sons, either when socially paired to their sons as the dominant pair on a territory or when the sons are acting as helpers to their mother (Cockburn et al. 2003). This suggests fairy‐wrens use extra‐pair reproduction to avoid close inbreeding. Furthermore, mother–son pairs show no behavioral interest in each other as potential mates (A. Cockburn, pers. obs.). However, whether the three assumptions underlying the inbreeding‐avoidance hypothesis outlined above all hold, and whether inbreeding avoidance extends to other levels of relatedness, is not known. Recently, Brouwer et al. (2017) explored multiple possible explanations for extra‐pair reproduction across the fairy‐wren (Maluridae) family. In support of inbreeding‐avoidance, studies of four other fairy‐wren species reported higher infidelity when social partners are more closely related (splendid fairy‐wrens, Brooker et al. 1990; Tarvin et al. 2005; red‐winged, Brouwer et al. 2011; red‐backed, Varian‐Ramos and Webster 2012; purple‐crowned, Kingma et al. 2013). However, these conclusions are all based on estimates of relatedness from microsatellite markers, rather than pedigree‐based knowledge of identity of close relatives. Furthermore, despite invoking the inbreeding‐avoidance hypothesis, these analyses do not assess inbreeding depression for offspring traits (with the exception of Kingma et al. (2013) finding evidence of hatching failure) or the implications of infidelity for inbreeding levels in the offspring.

Our aims in this study were fivefold. Firstly, we used 26 years of multigenerational genetic pedigree data to quantify the frequency of inbreeding in superb fairy‐wrens, and to identify particular routes by which it might occur. We then tested the three assumptions of the hypothesis that extra‐pair reproduction facilitates inbreeding avoidance by: (1) assessing inbreeding depression in two offspring traits, nestling mass, and fledgling survival; (2) testing whether females who were socially paired to a relative were more likely to be unfaithful, extending the previous documentation of 100% infidelity in mother‐son pairings (Cockburn et al. 2003) to consider all possible levels of kinship between social partners. Then, (3) we quantified the overall effects of infidelity on the probability of offspring being inbred, testing whether extra‐pair offspring were less likely to be inbred than within‐pair offspring, and comparing observed levels of inbreeding with those that would have occurred had females always been entirely faithful. Finally, we assessed the impact of the social environment on each of these four aspects, by considering whether effects were mediated by the number of helpers in all analyses.

## Materials and Methods

### STUDY SYSTEM

The colour‐banded population of superb fairy‐wrens (*Malurus cyaneus*) in and around the Australian National Botanic Gardens, Canberra, Australia (35°16 S, 149°06 E) has been intensively monitored since 1988 (Cockburn et al. 2003). The study site measures ∼60 ha, contains 60–90 territories/year, and is surrounded by unmonitored superb fairy‐wren territories; the study population is thus a sample of a much larger population with free movement across its boundaries. In this article, we use data from years 1988–2013.

All study population birds were censused throughout the year (Cockburn et al. 2003), with data collected on group composition, social pairings, fates, and reproductive performance of individuals. Females can successfully raise up to three broods in a single season (between August and March each year), with each brood containing 3–4 young (Cockburn et al. 2008c). However, because predation rates are high, as many as eight clutches may be initiated in a season (Cockburn et al. 2008c). Nestlings were banded 5–8 days after hatching. A blood sample was taken at the same time, and microsatellite genotyping was used to assign parentage to all individuals (Double et al. 1997); for paternity assignment details see the Supplementary Information (SI).

Since we tested the association between the number of helpers and EPP rates, it is worth noting that the large majority of extra‐pair paternity is extra‐group (i.e., involving a male on a different territory, in a different social group). Helpers gain little paternity within their social group, although many gain substantial reproductive success through extra‐group matings (Double and Cockburn 2003). In particular, helpers only gain within‐group paternity when their mother is no longer the breeding female, because she has either died or divorced her social partner to move to another territory, and so has then been replaced as breeder by an unrelated female (Cockburn et al. 2003). Furthermore, if the breeding female is socially paired with her son, all other helpers on the territory will most likely also be her sons (Cockburn et al. 2008b), leaving the female with no outbred mating opportunities on the territory.

### SECTION 1: PEDIGREE RECONSTRUCTION AND QUANTIFYING LEVELS OF INBREEDING

We used eight exceptionally polymorphic microsatellite loci and a stepwise process to assign paternities while taking into account the structure of our population; this allows identification of sires with near 100% certainty. Further details are provided in the SI. Using the parentage data, we constructed a multigenerational pedigree for individuals sampled between 1988 and 2013: this pedigree had maximum depth of 15 generations. We estimated inbreeding coefficients (*f*) from the pedigree for individuals for whom the identities of both genetic parents and at least one grandparent were known (*n* = 4431). Note that inbreeding between distant relatives could have been underestimated for individuals with less complete pedigree data. We therefore provide details of the effect of restricting to higher numbers of known grandparents on the sample sizes and inbreeding rates in the SI (Table S1).

For each social pair (i.e., for each territory, the breeding female, and the dominant male—always the oldest male on the territory), we calculated a kinship coefficient (*k_SOC_*), and for each female‐EP male pair that produced extra‐pair offspring (EPO), we also calculated a kinship coefficient (*k_EP_*). The kinship coefficient between two individuals is defined as the probability that homologous alleles sampled from two individuals are identical by descent (Wright 1922), and is equal to the inbreeding coefficient of offspring that would be produced by these individuals. Throughout, we distinguish between the “genetic father,” meaning the male who sired a particular offspring, and the “social father,” meaning the male who was dominant on the territory at the time that the offspring was hatched, and who may or may not have been the genetic father.

The variables fitted in the statistical models described below varied depending on the model, thus sample sizes for individual models varied and are given alongside the model results. All analyses were carried out in *R* version 3.3.1 (Development Core Team 2011). See SI for general information on individual/parent numbers in our main dataset.

### SECTION 2: INBREEDING DEPRESSION

We estimated inbreeding depression in nestling mass and in fledgling survival.

#### Inbreeding depression in nestling mass

We fitted a linear‐mixed effects animal model using the R package *ASReml‐R* version 3 (Butler et al. 2009), with *nestling mass* (continuous) as the response variable, with Gaussian errors. An animal model (i.e., incorporating an additive genetic effect; Kruuk 2004) was fitted to avoid any potential bias of estimates of inbreeding depression by not accounting for heritable genetic effects (Becker et al. 2016), and to provide an estimate of the heritability of nestling mass. Significance of fixed effects was assessed using Wald statistics (with critical level of *P* < 0.05). **Fixed effects**: The *inbreeding coefficient* of each individual was fitted to test for potential inbreeding depression. We also fitted: *number of helpers* (as a three‐level factor: 0, 1, and 2+; where 2+ level consisted mainly of two helpers with some pairs assisted by three or four helpers; Kruuk et al. 2015); *brood size* (the number of nestlings, 3–5), to account for the variation in the amount of care provided to the individual nestlings; and *sex of nestling* (male, female), to account for differences in size between males and females. *Nestling age* at measurement (continuous, in days; as a quadratic function) was fitted because pragmatic considerations meant that nestlings were weighed at different ages (days 5–8) and hence at different stages of their development. We fitted a two‐level factor (pre‐1992, 1992+) to account for the introduction of a new weighing protocol in 1992, which changed the time of day at which nestlings were weighed (Kruuk et al. 2015). **Random effects**: We fitted *nest ID* to account for any similarities across multiple offspring from the same brood; an *additive genetic effect* (with covariance structure determined by the pedigree) to test for covariance between relatives (Kruuk 2004); and a multilevel factor of *cohort* to represent interannual variation (1988–2013: the “2013” cohort incorporates nestlings from August 2013 through to March 2014 etc.). Finally, we represented intraannual temporal variation across the breeding season by fitting a multilevel factor of *hatch date interval* (split into 12 two‐week intervals, between 23 September and 15 March).

The above model was run using the R package *ASReml‐R*, as the response required Gaussian errors. All following models were run using the R package *MCMCglmm* (Hadfield 2010) to allow for binomial errors. For all the *MCMCglmm* models the effective sample sizes for specific parameters varied due to autocorrelation, but we ensured that they were always above 1000. We considered terms to be statistically significant based on 95% CIs (credible intervals) not spanning 0 and *pMCMC* values (the number of simulations greater or smaller than 0 corrected for number of *MCMC* samples) calculated by *MCMCglmm* being <0.05. Details of model settings, such as the number of iterations, burn‐in, thinning interval, and priors for each model can be found in the SI.

#### Inbreeding depression in survival

We investigated survival from fledging to independence (from 12 to 41 days, see the SI for details on how these bounds were chosen). We ran two generalized linear‐mixed effects animal models using the *MCMCglmm* package. We first tested whether inbreeding affected survival, and then investigated whether any effect of inbreeding acted through body mass, by including mass as a covariate in the analysis. *Survival* was modeled as a binary (0/1) response variable, with binomial errors. **Fixed effects**: *Inbreeding coefficient, number of helpers, brood size*, and an *individual's sex* were fitted as described above (with *nestling mass* as an additional covariate in one of the models). **Random effects**: *Nest ID, additive genetic effect, cohort*, and *hatch date interval* were fitted as described above.

We added in an interaction between the inbreeding coefficient and the number of helpers, to test whether helpers could mitigate the effects of inbreeding depression (see SI).

### SECTION 3: EFFECTS OF KINSHIP BETWEEN SOCIAL PARTNERS ON INFIDELITY

We tested whether patterns of EPP were associated with either kinship between social partners (*k_SOC_*) and/or social environment, specifically the number of helpers at the nest. We used the proportion of EPO in each brood (as an index of infidelity) to investigate factors affecting a female's likelihood of producing extra‐pair young.

It is possible that inbreeding depression in early embryo/nestling survival could bias later estimates of the extent of EPP in pairs where social partners are related. If all WPO die due to inbreeding depression, the clutch will appear to be composed entirely of EPO and rates of EPP will be overestimated (Reid 2015; Reid et al. 2015b). To assess whether such “selective disappearance” affected our estimates, we tested whether kinship between the social partners affected clutch size and/or survival of nestlings prior to measurement age. We found that *k_SOC_* was not associated with either clutch size or survival to measurement (Table S2). (Note that this analysis also provided a test of inbreeding depression in early survival, but that this test was indirect; highly accurate assessment of early inbreeding depression is difficult. See SI for details.)

When assessing EPP levels, cases of mother–son pairings required special consideration. As described above, both behavioral and genetic analyses indicate that mothers and their sons never mate, even when socially paired, suggesting strong inbreeding avoidance in these pairings. We therefore ran two versions of our models: (a) using all available data; and (b) excluding mother–son pairings. This allowed us to test whether the results were disproportionately affected by the special case of mother–son pairings, without restricting exploration of the effects of kinship and the social environment beyond the mother–son pairings.

To test the effect of kinship between social partners on infidelity we fitted binomial generalized linear‐mixed models in *MCMCglmm*, with the proportion of EPO in a brood (defined by the numbers of extra‐ *vs*. within‐pair offspring) as a response variable, and binomial errors; these models were by definition fitted at the level of the brood rather than individual nestlings. **Fixed effects**: *Kinship* (continuous) was fitted to test whether the probability of offspring being sired by an extra‐pair male varied with the kinship between the female and her social mate (*k_SOC_*). The *number of helpers* (0, 1, and 2+) was fitted to test whether the social environment affected the probability of extra‐pair reproduction. Additionally, the *mother's age* and the *social father's age* (two‐level factors: one‐year‐old, older) were fitted to account for potential effects of differences in experience. **Random effects**: *Mother ID* and *social father ID* were fitted to account for the multiple observations on specific females and males (social fathers). *Cohort* was fitted as above.

We then fitted both of the models of EPP rates with an interaction between the *k_SOC_* and the number of helpers, to test for the role of social environment (see SI).

### SECTION 4: EFFECTS OF INFIDELITY ON INBREEDING

This final section tested the consequences of EPP for the probability of offspring being inbred. Due to the high proportion of zeros (94.5%) among the estimated inbreeding coefficients, the inbreeding coefficient did not fit any standard distribution for a continuous covariate. We therefore used a two‐step process: first we fitted a model with a binomial response of whether an individual offspring was inbred (*f* > 0) or not (*f* = 0), and then we fitted a model with a continuous response, but only considering inbred individuals (*f* > 0) (see Huisman et al. 2016 for a similar two‐stage analysis).

The models were fitted excluding mother–son pairings to avoid any bias stemming from those special cases. Additionally, because the number of helpers affects rates of EPP (Cockburn et al. 2016 and references therein, and see below), we did not fit the number of helpers in these models, given the potential confounding effects between the two.

**Step 1**. We fitted a binomial generalised linear‐mixed model using *MCMCglmm*, with the *inbreeding status* of every offspring as a response (two‐level factor: inbred *vs*. outbred, where an inbred individual had *f* > 0). **Fixed effects**: We fitted *within‐pair status*, that is whether an offspring was the result of within‐pair (WP) or extra‐pair (EP) reproduction, as a two‐level factor. **Random effects**: *Nest ID* and *cohort* were fitted as above.
**Step 2**. We tested what determined the magnitude of the inbreeding coefficient amongst those nestlings that were inbred, that is had *f* > 0. We fitted a linear mixed model using *MCMCglmm*, with log‐transformed inbreeding coefficient as the response variable, and Gaussian errors. We used only the inbred individuals (*f* > 0) in this model. **Fixed effects**: We fitted *within‐pair status* (a two‐level factor: WP *vs*. EP). **Random effects**: *Nest ID* and *cohort* were fitted as above.

As a final step, we asked what level of inbreeding would have occurred if there had never been any extra‐pair reproduction. To do this, we constructed an artificial social “faithful” pedigree to represent the relatedness patterns that would have occurred had all females always been faithful and their social mates were always the genetic fathers of all their offspring. Comparing the inbreeding coefficients that would have resulted if this pedigree were real with the actual observed inbreeding coefficients allowed us to judge the overall impact of infidelity on inbreeding in the population.

In order for the *MCMCglmm* models to run satisfactorily, it was necessary to truncate the latent variables for models in this section (J. Hadfield, pers. comm.); see SI for details.

## RESULTS

### SECTION 1: PEDIGREE RECONSTRUCTION AND LEVELS OF INBREEDING

Kinship between superb fairy‐wren social partners (*k_SOC_*) ranged from zero to 0.25, with the latter category consisting entirely of pairings between mothers and sons; the mean *k_SOC_* across 863 unique social pairings was 0.0129 (*median* = 0). Only 10.5% of social pairings were incestuous (*k_SOC_* > 0), with mother–son pairings accounting for 4.2% of all social pairings (Table 1A).

**Table 1:** Distribution of kinship between social partners and of inbreeding coefficients

(A) Kinship between social partners (brood level)		Broods % (*n*)
All: $k_{soc}>0$		10.5% (183/1745)
High: $k_{soc}\geq 0.25$		4.2% (73/1745)
Moderate: $0.125\leq k_{soc}<0.25$		0.7% (12/1745)
Low: $k_{soc}<0.125$		5.6% (98/1745)
Male social partner	Female social partner	Broods % (*n*)
**High**: $k_{soc}\geq 0.25$		
son	Mother	4.2% (73/1745)
**Moderate**: $0.125\leq k_{soc}<0.25$		
Paternal half‐brother	Paternal half‐sister	0.2% (4/1745)
Maternal half‐brother	Maternal half‐sister	0.2% (4/1745)
Grandson	Maternal grandmother	0.2% (4/1745)
(B) Inbreeding coefficient (individual level)		Individuals % (*n*)
All: f>0		5.5% (245/4431)
High: f≥0.25		0.0% (0/4431)
Moderate: 0.125≤f<0.25 †		0.3% (14/4431)
Low: f<0.125		5.2% (231/4431)
Male parent	Female parent	Individuals % (n)
**Moderate**: 0.125≤f<0.25 †		
Paternal half‐brother	Paternal half‐sister	0.09% (4/4431)
Maternal half‐brother	Maternal half‐sister	0.02% (1/4431)
Grandson	Paternal grandmother	0.05% (2/4431)
Grandson	Maternal grandmother	0.1% (5/4431)
Paternal grandfather	Granddaughter	0.05% (2/4431)

† For consistency with (A), we refer to “moderate” inbreeding as 0.125≤f<0.25, but in practice all individuals in this category were *f* = 0.125.

(A) Percentage of broods for which the social parents were relatives (*k_SOC_* > 0) (out of *n* = 1745 broods), then split into high, moderate, or low levels of *k_SOC_*, and followed by details of individual cases where *k_SOC_* ≥ 0.125; and (B) percentage of inbred offspring, considering the inbreeding coefficient (*f* > 0) at the individual level (out of *n* = 4431 individuals), then split into high, moderate, or low levels of *f*, followed by details of individual cases where *f* ≥ 0.125.

Inbreeding appeared rare: of the 4431 offspring with at least one grandparent known, only 5.5% were inbred (i.e., had *f* > 0) (Table 1B). There were no individuals with *f* = 0.25 (as would result from reproduction between a parent and offspring or from a full‐sibling pairing). The maximum inbreeding coefficient observed was *f* = 0.125, and occurred *via* a variety of routes (Table 1B). Tighter pedigree restrictions lead to an increased overall frequency of inbreeding, due to tighter restrictions generally resulting in the exclusion of “outbred” (*f* = 0) individuals (Table S1). This increase was particularly prominent when maternal grandparents were included in the restriction: a requirement of at least three known grandparents corresponded to a frequency of inbred individuals of 18.2%.

### SECTION 2: INBREEDING DEPRESSION ON NESTLING MASS AND SURVIVAL

**Figure 1:**
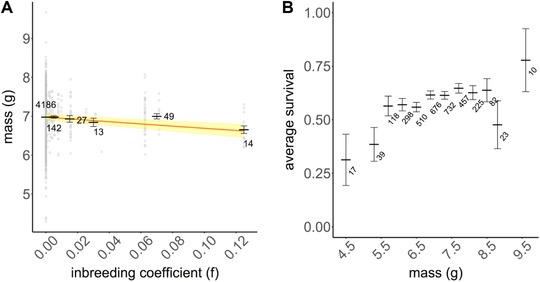
(A) Effects of inbreeding coefficient (*f*) on nestling mass. Mass was corrected for the change in protocol in 1992 and for the age of the nestling at measurement, with mean mass at day 7 being presented. Gray open circles represent the raw data, black dashes show means of data grouped into bins (0, between 0–0.01 noninclusive of bounds, then 0.01–0.02, 0.02–0.04, 0.06–0.08, 0.12–0.13 with lower bound inclusive) with the group sample sizes indicated next to the groups (total *n* = 4431), and with error bars representing standard errors. The solid orange line represents the predictions from a linear‐mixed effects model, aligned with the intercept of the raw data, with shading around the line showing standard errors. (B) Effects of nestling mass on survival from fledging to 41 days. Dashes represent mean survival of individuals with nestling mass binned (3.9–5.1, then every 0.4, till 8.7–9.0, 9.0–10.2, lower bound inclusive; note that bins at the extremes are wider) with error bars showing standard errors and group sample sizes indicated next to the groups (total *n* = 3187).

Nestling mass declined with inbreeding coefficient, providing evidence for inbreeding depression (Table 2, Fig. 1A): the mean observed mass for nestlings with *f * = 0.125 was ∼10% lower than the mean observed mass for nestlings with *f* = 0 (see Table S9 for predicted nestling mass across different *f* values). In line with previous results (Kruuk et al. 2015), we also found that: males were heavier than females; nestlings from smaller broods were heavier than those from larger broods; those from broods assisted by helpers were heavier than those from unassisted broods; and, unsurprisingly, nestling mass increased with the age at which they were measured (Table 2). Additionally, the heritability of nestling mass was estimated as 18.9% (±3.2% SE). However, the social environment had no effect on the realization of inbreeding depression (*P* = 0.98; Table S3A).

**Table 2:** Test for inbreeding depression

	**Nestling mass (g)**		**Survival from fledging to 41 days**			
			(B)		(C)	
	(A)		With mass		Without mass	
			**Posterior mean**		**Posterior mean**	
**Fixed effects**	**Estimate (SE)**	***P***	**(95% CI)**	***P***	**(95% CI)**	***P***
Intercept	−3.83 (1.21)	**0.008**	−0.92 (−3.02, 1.18)	0.364	1.41 (0.14, 2.73)	**0.035**
1992 (1992 + , pre‐1992)		<**0.001**				
pre‐1992	0.62 (0.12)					
Nestling age	2.16 (0.35)	<**0.001**				
Nestling age^2^	−0.09 (0.03)	<**0.001**				
Brood size	−0.05 (0.02)	**0.012**	−0.17 (−0.45, 0.13)	0.257	−0.20 (−0.48, 0.11)	0.194
Sex (female, male)		<**0.001**				
Male	0.15 (0.02)		−0.08 (−0.38, 0.18)	0.595	−0.02 (−0.30, 0.26)	0.881
Mass			0.33 (0.07, 0.55)	**0.004**		
Helpers (0, 1, 2 + )		<**0.001**				
1 helper	0.09 (0.04)		0.26 (−0.24, 0.73)	0.278	0.29 (−0.16, 0.78)	0.223
2+ helpers	0.20 (0.04)		0.33 (−0.20, 0.93)	0.252	0.40 (−0.14, 0.91)	0.146
Inbreeding coefficient	−3.64 (1.26)	**0.004**	−6.92 (−27.45, 13.83)	0.497	−5.33 (−24.35, 16.00)	0.590
**Random effects**	**Variance (SE)**		**Posterior mean**		**Posterior mean**	
			**(95% CI)**		**(95% CI)**	
Nest ID	0.23 (0.01)		6.53 (4.68, 8.76)		6.37 (4.48, 8.31)	
Hatch date	0.01 (0.01)		2.54 (0.60, 5.78)		2.67 (0.54, 5.89)	
Cohort	0.01 (0.01)		0.30 (8.91^−5^, 0.70)		0.32 (2.27^−5^, 0.75)	
Additive genetic effect	0.10 (0.02)		1.54 (0.17, 3.10)		1.48 (0.22, 3.00)	
Residual variance	0.19 (0.01)		n/a		n/a	
Sample size	4167		3187		3200	

Effects of inbreeding coefficient *f* on (A) nestling mass; and on survival from fledging to 41 days fitted (B) with nestling mass (corrected for change in protocol in 1992 and for nestling age at measurement) included as a covariate, and (C) without nestling mass included. There was no support for an interaction between the inbreeding coefficient and number of helpers, thus the interaction was dropped from the models and simple models are presented above; for models with interaction see Table S3. (Note that the precise form of output differs for the *ASReml‐R* model in (A) versus the *MCMCglmm* models in (B)/(C).)

In contrast, there was no evidence of inbreeding depression for survival, whether or not nestling mass was included in the model: the probability of a fledgling surviving to independence was not affected by its inbreeding coefficient. Heavier individuals did survive better (Fig. 1B, Table 2), but none of the other variables modeled were associated with changes in survival (Table 2). As above, we found no statistical support for an interaction between the effect of inbreeding on nestling mass and the number of helpers (Table S3).

### SECTION 3: EFFECTS OF KINSHIP BETWEEN SOCIAL PARTNERS ON THE LEVELS OF EXTRA‐PAIR REPRODUCTION

Out of the 4431 individuals with known parents and at least one known grandparent, 2704 (61%) were extra‐pair, and out of the total 1745 broods, 1445 (82.8%) had at least one extra‐pair offspring.

**Figure 2:**
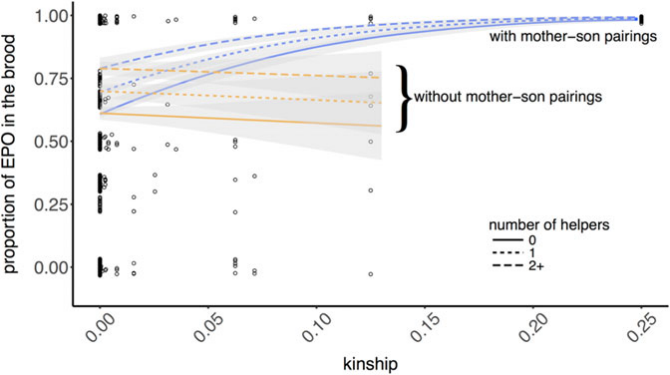
Effects of kinship between the social male and female on the proportion of extra‐pair offspring in the brood. Open circles represent raw data, which has been jittered to aid visualization. Data are presented with mother‐son pairings (blue lines), as well as without the broods produced by females socially paired to their sons (“without mother‐son pairings”; orange lines). The lines represent model predictions from generalized linear‐mixed effects models, split by the number of helpers (0, 1, and 2+) to emphasize the impact of helpers, with shading around the lines showing standard errors.

Considering the full dataset including the mother–son pairings, the proportion of EPO in the brood increased with increasing kinship, *k_SOC_* (Fig. 2, Table 3A). However, *k_SOC_* had no effect on EPO frequency when the mother–son pairings were removed (Fig. 2, Table 3B), indicating that its effect on infidelity rates was driven by the special case of mother–son pairings, where infidelity was 100%. Outside of the mother–son pairings, the mean percentage of EPO per brood was 73.3%. The proportion of EPO also increased with increasing helper number, regardless of whether mother–son pairings were included in the model or not. However, the effects of *k_SOC_* were not modulated by the number of helpers (Table S4). Mother's age and social father's age also had no effect in either model (Table 3).

**Table 3:** Effects of kinship between the social male and female and the effects of helpers on the proportion of extra‐pair offspring in the brood

	Proportion of extra‐pair offspring in the brood			
	(A)		(B)	
	With mother–son		Without mother–son	
Fixed effects	Posterior mean (95% CI)	*P*	Posterior mean (95% CI)	*P*
Intercept	0.47 (0.11, 0.84)	**0.017**	0.49 (0.13, 0.88)	**0.009**
Mother age (1yo, older)				
older	0.16 (−0.12, 0.42)	0.248	0.13 (−0.15, 0.39)	0.356
Social father age (1yo, older)				
older	−0.16 (−0.55, 0.20)	0.400	−0.13 (−0.53, 0.25)	0.522
Helpers (0, 1, 2+)				
1 helper	0.53 (0.24, 0.82)	**0.004**	0.55 (0.23, 0.81)	**0.002**
2+ helpers	1.17 (0.78, 1.55)	<**0.001**	1.17 (0.82, 1.58)	<**0.001**
Kinship	18.69 (13.65, 24.29)	<**0.001**	−1.19 (−11.04, 9.41)	0.807
Random effects	Posterior mean (95% CI)		Posterior mean (95% CI)	
Mother ID	0.73 (0.33, 1.15)		0.69 (0.30, 1.09)	
Social father ID	0.71 (0.33, 1.07)		0.72 (0.40, 1.12)	
Cohort	0.02 (5.36^−09^, 0.06)		0.02 (9.73^−09^, 0.06)	
Residual variance	1.85 (1.35, 2.33)		1.82 (1.38, 2.36)	
Sample size	1473		1421	

Models were run **(A)** on all data, including mother–son pairings; and **(B)** excluding mother–son pairings and any offspring produced by females socially paired to their sons.

### SECTION 4: EFFECTS OF INFIDELITY ON THE PROBABILITY OF OFFSPRING BEING INBRED

Kinship between females and their extra‐pair partners (*k_EP_*) ranged from zero up to 0.125 (Table 1A), with the highest value representing three extra‐pair pairings that occurred between grandmothers and grandsons, and one extra‐pair pairing between granddaughter and grandfather; the mean *k_EP_* across 1197 unique extra‐pair pairings was 0.0012 (*median*  =  0). Infidelity reduced inbreeding: if all females had been faithful to their social partners throughout the study period, 10.4% of all individuals in the population would be inbred (*f* > 0) compared to the observed 5.5% (Table 4; Fisher's exact test: *P* < 0.001). Excluding mother–son pairings, these frequencies become 9.3% *vs*. 4.5% (Fisher's exact test: *P* < 0.001).

**Table 4:** Numbers and percentages of inbred and outbred within‐ and extra‐pair offspring

	Inbred	Outbred	Row total
Within‐pair	130	1597	1727
	7.5%	92.5%	
Extra‐pair	115	2589	2704
	4.3%	95.8%	
Observed total	245	4186	4431
	5.5%	94.5%	
If faithful total	459	3972	4431
	10.4%	89.6%	

Figures for inbred and outbred offspring if all females were always faithful to their social partners, that is with no extra‐pair offspring present in the population (“if faithful total”) are also given for comparison. Percentages are presented per row and rounded to 1 decimal place. Any individual with inbreeding coefficient *f* > 0 was classified as inbred.

Table 4 shows the frequency of inbred (*f* > 0) *vs*. outbred (*f* = 0) individuals in relation to their extra‐pair *vs*. within‐pair status: there were more inbred WPO than EPO (Fisher's exact test: *P* < 0.001). Moreover, this was the case regardless of pedigree restrictions applied (Table S5). The mixed model confirmed that EPO were less likely to be inbred than WPO (Table 5), while the number of helpers had no effect on inbreeding status (Table S8). These results were also consistent across different pedigree restrictions (Table S6). Furthermore, the second step of this analysis showed that amongst inbred offspring (*f* > 0), EPO had lower inbreeding coefficients than WPO (Table 6).

**Table 5:** Effects of within‐pair status of an offspring (whether it was within‐pair, WP, or extra‐pair, EP) on the offspring's inbreeding status (whether it was inbred, with *f* > 0, or outbred, with *f* = 0), using a binomial‐mixed model run in *MCMCglmm*

	Inbreeding status of an individual	
	(inbred *vs* outbred)	
**Fixed effects**	**Posterior mean (95% CI)**	***P***
Intercept	−9.83 (−10.80, −8.91)	<**0.001**
Within‐pair status (EP, WP)		
WP	1.36 (0.70, 1.98)	<**0.001**
**Random effects**	**Posterior mean (95% CI)**	
Nest ID	25.12 (21.11, 29.51)	
Cohort	2.55 (0.46, 5.23)	
Sample size	4283	

The model was run without mother–son pairings.

**Table 6:** Effects of within‐pair status of an inbred (*i.e*., *f* > 0) offspring (whether it was within‐pair, WP, or extra‐pair, EP) on the offspring's level of inbreeding *f* (continuous), using a linear mixed model run in *MCMCglmm*

	Inbreeding level of an individual	
	(0 <f≤0.125)	
**Fixed effects**	**Posterior mean (95% CI)**	***P***
Intercept	−4.99 (−5.70, −4.26)	<**0.001**
Within‐pair status (EP, WP)		
WP	0.50 (0.17, 0.77)	<**0.001**
**Random effects**	**Posterior mean (95% CI)**	
Nest ID	4.02 (2.86, 5.17)	
Cohort	1.63 (0.29, 3.32)	
Residual variance	0.15 (0.11, 0.18)	
Sample size	245	

The model was run without mother–son pairings.

## DISCUSSION

Our study explored the associations between, and the effects of, inbreeding, kinship, and infidelity in a wild population of a cooperatively breeding bird. We found some support for each of the three key assumptions outlined in the Introduction as necessary for the hypothesis that extra‐pair reproduction is driven by inbreeding avoidance. Thus we found **(1)** evidence of inbreeding depression in nestling mass; **(2)** that increased kinship between social partners was associated with a higher frequency of extra‐pair offspring in the brood; and **(3)** that extra‐pair offspring were less likely to be inbred than within‐pair offspring, and when inbred, they had lower inbreeding coefficients than within‐pair offspring. However, detailed analysis revealed that kinship‐infidelity results were context‐specific: the frequency of extra‐pair offspring only increased with kinship when considering mother–son pairs. Given that only 4.2% of broods had mother–son pairs as social parents, our results suggest that the inbreeding‐avoidance hypothesis cannot explain the widespread occurrence of extra‐pair reproduction in our system. We also found no evidence that any of these aspects were affected by the social environment. We discuss each of these points in turn below.

### INBREEDING AND INBREEDING DEPRESSION

Both social pairing between close relatives and moderate‐level inbreeding were rare in our population: only 4.9% of pairs had *k_SOC_* ≥ 0.125 and only 0.3% of individuals had *f* between 0.125 and 0.25 (Table 1). There was no high‐level inbreeding (*f* ≥ 0.25). The *overall* frequency of incestuous pairings and of inbreeding events were 10.5% (*k_SOC_* > 0) and 5.5% (*f* > 0), respectively. These overall frequencies increased with tighter restrictions on the pedigree to 24.6% incestuous pairs and 18.2% inbred offspring for 3+ known grandparents, and to 28.5% incestuous pairs and 21.5% inbred offspring with four grandparents known (Table S1). The increased frequency presumably reflects, in part, the exclusion of pairs/individuals erroneously assigned *k_SOC_* = 0 and *f* = 0 because their ancestry information was not sufficient to identify lower levels of relatedness/inbreeding: without a perfectly complete pedigree, inbreeding between distant relatives may not be detected, leading to an underestimation of the overall occurrence of inbreeding. However, the steep increase beyond 2+ known grandparents is likely predominately due to biasing the dataset toward females who have dispersed shorter distances from their natal territory, as our ability to sample all grandparents was often restricted to these females. These short‐dispersing females will be more likely to encounter male relatives as partners than females dispersing over longer distances (in superb fairy‐wrens female dispersal is obligatory (Mulder 1995)). Our results therefore indicate that inbreeding and social pairing between close relatives were rare in this population. The overall levels detected were nevertheless comparable to several other bird populations (reviewed in Kruuk et al. 2002). For instance, in a British great tit population, only 1.3% of pairings were between first‐ or second‐order relatives (Szulkin et al. 2007), whereas in our superb fairy‐wren population it was 4.9% of pairings (high/moderate *k_SOC_*, Table 1A). In contrast, the Mandarte Island song sparrows have substantially higher frequency of closely related pairings: 21.4% of pairings were between first‐ or second‐order relatives (Reid et al. 2015c).

We note also that theory suggests that under certain conditions father–daughter pairings may be more likely than mother–son pairings (Waser et al. 1986). However, in superb fairy‐wrens, obligate female dispersal means that females never pair with their social fathers and so the only feasible social pairing of close/familiar relatives in this system are mother–son pairings (all of *k_SOC_*  =  0.25 cases here). While a female could conceivably disperse and pair/mate with her extra‐group sire, we have never observed this. Such pairings may be unlikely in this system due to the dispersal distances typically being too large to facilitate father–daughter contact. As a result, we do not see offspring resulting from pairings between females and their fathers.

We found evidence for inbreeding depression in nestling mass, of magnitude comparable to other studies (e.g., Soay sheep on the islands of St. Kilda (Berenos et al. 2016)). However, although nestling mass positively affected survival, there was no evidence for inbreeding depression in survival. Inbred offspring with *f* = 0.125 surviving to fledging were on average 6.5% lighter than outbred offspring (*f* = 0.125: mean observed nestling mass ∼6.6 g; *f* = 0: mean observed nestling mass ∼ 7.0 g). The average survival of fledglings with nestling mass 6.5–6.7 g was 54%, while for fledglings with nestling mass 6.9–7.1 g it was 58%. Therefore, all else being equal, a reduction in nestling mass should have translated into ∼4% reduction in survival for inbred fledglings (*f* = 0.125), but this was not evident from our survival models (in which estimates suggested inbreeding depression in survival, but 95% CIs spanned 0). Due to the rarity of inbreeding events between close relatives, the lack of statistical support for inbreeding depression in survival may be due, in part, to a lack of statistical power, but these calculations suggest that any reduction in survival within this period may not be large. We did not test for inbreeding depression in adult traits due to the low number of inbred adult birds. Thus detecting inbreeding depression may be easier in populations with higher levels of inbreeding. It may also be facilitated by use of high‐density genomic marker data, which can reveal variation in genome‐wide heterozygosity among individuals classified as *f* =  0 with a pedigree analysis (Huisman et al. 2016). Regardless, our analyses here do not indicate strong inbreeding depression in survival in this population.

Two further caveats are worth pointing out with regard to interpretation of the estimated occurrence of inbreeding depression. Firstly, it is of course difficult to ascertain how the level of inbreeding depression observed during the period of this study may compare to the severity of inbreeding depression in the past, and thus difficult to infer past selection pressures against inbred individuals. Second, while inbreeding depression of some form is necessary for inbreeding avoidance to provide a plausible explanation for extra‐pair paternity, it would need to be sufficiently strong for the adaptive benefits of inbreeding avoidance to counter any costs and any potential inclusive fitness benefits of inbreeding (Szulkin et al. 2013; Duthie and Reid 2016; Duthie et al. 2016a).

### INFIDELITY VARIATION WITH KINSHIP

Our analyses confirmed Cockburn et al.’s (2003) results: mother–son pairings resulted in absolute infidelity by the female, with no WPO produced by such pairings. These results appear robust to “selective disappearance” as we found no evidence for reduced clutch size and/or survival to measurement age, as would happen if EPO were more likely to survive to measurement than WPO due to inbreeding depression (Reid et al. 2015b). However, there was no evidence of inbreeding avoidance through extra‐pair reproduction in cases other than the mother–son pairs, who were the social parents of 4.2% of the observed broods. This, together with behavioral data indicating that all females seek extra‐pair copulations throughout the breeding season (Cockburn et al. 2016), suggests that the main reason for extra‐pair reproduction for the majority of the females in our study is *not* inbreeding avoidance.

The lack of a simple population‐wide relationship between *k_SOC_* and infidelity indicates that explanations for infidelity can be context‐dependent. Brouwer et al. (2017) recently showed that explanations for infidelity patterns in the fairy‐wren family vary depending on the level of analysis and the spatiotemporal scale used. In our study, only females in mother–son pairings avoided inbreeding through increased infidelity, and the hypothesis of inbreeding avoidance through EP reproduction was not supported for other females. Yet, studies typically investigate this hypothesis at a population‐level, often assuming that a simple relationship, or lack thereof, between kinship and infidelity is evidence for the existence of a population‐wide explanation for infidelity. Even across other species of Maluridae, several studies have shown higher infidelity in incestuous pairs (Brooker et al. 1990; Tarvin et al. 2005; Brouwer et al. 2011; Varian‐Ramos and Webster 2012; Kingma et al. 2013), but we note that where relatedness between social partners is assessed by genetic markers, it is not possible to distinguish certain types of relationships, such as mother–son pairings, for which different strategies may apply. Thus we suggest that care is needed when interpreting *overall* relationships between kinship and infidelity as evidence of a *general* inbreeding avoidance “strategy” applicable to all individuals in a population. Our data suggest that such relationships may vary and that very different patterns in a relatively small number of individuals, not necessarily representative of the whole population, could drive the results of an analysis.

Furthermore, evidence suggests that, in general, bird species can only recognise kin when there is strong contextual evidence of relationship, such as having been raised in the same nest (Nakagawa and Waas 2004; Ihle and Forstmeier 2013). It would seem unlikely that females are able to distinguish kin, beyond close/familiar relatives, from nonkin, and thus unlikely that they would actively allocate paternity based on kinship to a less‐closely related social partner. Close relatives, such as nuclear family members and familiar individuals (e.g., a female's offspring, nest mates), may present a special case that should potentially be considered separately from more distant relatives in these types of studies.

### INFIDELITY REDUCES INBREEDING

The levels of inbreeding we detected were low: 94.3% of our inbred individuals had inbreeding levels of *f* < 0.125. Yet, we found that EPO were more likely to be outbred than WPO, even after the exclusion of the mother–son pairings, and that amongst inbred offspring, EPO had lower inbreeding coefficients than WPO. These results demonstrate that differences in inbreeding status and inbreeding levels between WPO and EPO can arise even without a relationship between kinship (*k_SOC_*) and infidelity (we found none when mother–son pairings were excluded).

Due to the lack of a relationship between kinship and infidelity beyond the mother–son pairings, active mate choice for less‐related males seems unlikely to be explaining the difference in inbreeding status between WPO and EPO. What is driving this relationship? We suggest it is likely to be linked to the demographic and/or spatial structure of the population: females may be, on average, less closely related to males in another group, several territories away, than they are to their social partners. Potentially, this pattern could be linked to nonrandom formation and/or persistence of more‐related––than expected by chance––social pairs (Reid et al. 2015c), kin structure, that is relatedness between individuals varying with distance (Nakagawa and Waas 2004; Foerster et al. 2006), or constraints on mate availability (Duthie et al. 2016a). A more detailed analysis will be required to understand whether the spatial and/or temporal distribution of mates can explain the pattern we observe.

### ASSESSMENT OF THE INBREEDING AVOIDANCE EXPLANATION FOR EXTRA‐PAIR PATERNITY

At a first glance all three assumptions necessary for the hypothesis of inbreeding avoidance through EP reproduction––inbreeding depression, increase in EPP when social partners are related, and a reduction in inbreeding *via* infidelity––were met in our study system. However, dissecting the infidelity‐kinship relationship we found that there was no population‐wide pattern, and that the effect of kinship on infidelity was driven entirely by mother–son pairs. Looking beyond such pairings, the risk of mating with relatives appeared too low and the degree of kin recognition required too fine‐scale for inbreeding avoidance to serve as a plausible explanation for EPP in this system. Given that only 0.7% of pairings involved moderate kinship levels (0.125 ≤ *k_SOC_* < 0.25), that is at a level that could be relevant to female choice, it seems highly unlikely that a complex behaviour such as extra‐pair reproduction would occur in the vast majority of breeding events just to mitigate this small risk of inbreeding: behavioral evidence suggests that females always seek EP copulations *via* extra‐territorial forays (Cockburn et al. 2016), and 82.8% of broods have at least one EP offspring. It therefore seems likely that other explanations for extra‐pair reproduction, such as choice of a preferred male in light of restricted social partner choice (e.g., choosing males with earlier moult date (Dunn and Cockburn 1999; Cockburn et al. 2008a)), are more plausible. Our study therefore illustrates the need to test all components of the hypothesis of inbreeding avoidance through EP reproduction explicitly: specifically, relationships between kinship and infidelity, as well as differences between WPO and EPO need to be assessed in detail.

### SOCIAL ENVIRONMENT

The social environment influenced patterns of infidelity, but not the occurrence or consequences of inbreeding. It had a strong influence on infidelity rates, with females assisted by helpers producing more EPO, predominantly sired by males outside of the entire social group (95% of the EPO were extra‐group offspring; G.K. Hajduk, unpubl. data); in particular, for reasons that are not entirely clear, the number of unrelated helpers is the best predictor of levels of extra‐group paternity (G.K. Hajduk, unpubl. data). However, the presence of helpers did not affect the probability of offspring in a nest being inbred (Table S8). Furthermore, while nestlings from broods assisted by helpers were heavier than those from unassisted broods (as in Kruuk et al. 2015), there was no evidence that the presence of helpers affected the occurrence or magnitude of inbreeding depression (Table 2; Table S3). These results fit with the general impression that it may be difficult to detect interactions of inbreeding depression with environmental conditions in natural populations (Pemberton et al. 2017). However, it is possible that we were simply unable to find support for interactions with inbreeding depression in our study population due to the rarity of inbreeding events.

## Conclusions

Our study used multigenerational pedigree data from a long‐term individual‐based study of a wild population to investigate patterns of inbreeding and inbreeding depression, as well as causes and consequences of extra‐pair reproduction. We showed that the relationship between kinship and infidelity may be complex, context‐dependent and not necessarily population‐wide, and that results can be easily driven by a small sample of individuals––in this case mother–son pairings. Additionally, we showed that within‐pair and extra‐pair offspring can differ in their probability of being inbred, even when infidelity occurs for reasons apparently unrelated to inbreeding avoidance. Furthermore, the social environment affected infidelity rates, but did not affect the probability of offspring being inbred or inbreeding depression. Overall, our study demonstrates how the social system of a population may affect mating patterns and their consequences in multiple complex ways, and also illustrates the value of long‐term pedigree data for providing insights into core aspects of evolutionary biology.

## CONFLICT OF INTEREST

The authors declare no conflict of interests.

Associate Editor: E. Derryberry

Handling Editor: M. Servedio

## Supporting information


**Table S1**. Pedigree restrictions on number of known grandparents affected not only the sample size, but also the rates of incestuous pairings between social partners (*k_SOC_* > 0) and inbreeding (*f* > 0).
**Table S2**. Effects of kinship between the social male and female and the effects of helpers on (a) clutch size, and (b) hatchability/survival of offspring to measurement age. Output from *MCMCglmm* models: sample sizes are number of broods across 26 cohorts.
**Table S3**. Test for inbreeding depression and the effects of social environment on the magnitude of inbreeding depression (an interaction between the inbreeding coefficient and number of helpers).
**Table S4**. Effects of kinship between the social male and female and the effects of helpers on the proportion of extra‐pair offspring in the brood, including an interaction between *k_SOC_* and helpers.
**Table S5**. Effects of pedigree restrictions on numbers and percentages of inbred and outbred within‐ and extra‐pair offspring.
**Table S6**. Pedigree restrictions: effects of within‐pair status of an individual (whether it was within‐pair, WP, or extra‐pair, EP) on the individual's inbreeding status (whether it was inbred, with *f* > 0, or outbred, with *f* = 0), using binomial mixed models run in *MCMCglmm*.
**Table S7**. Pedigree restrictions: effects of within‐pair status of an individual (whether it was within‐pair, WP, or extra‐pair, EP) on the individual's inbreeding status (whether it was inbred, with *f* ≥ 0.0625, or outbred, with *f* < 0.0625), using binomial mixed models run in *MCMCglmm*.
**Table S8**. Effects of social environment ‐ number of helpers – on the probability of offspring being inbred.
**Table S9**. Predicted nestling mass (g) for increasing levels of inbreeding (standard errors in brackets).
