# Supplementary material for: Inbreeding, inbreeding depression, and infidelity in a cooperatively breeding bird
Source: Evolution. 2018 Jul 5;72(7):1500–14. doi: 10.1111/evo.13496 (PMC6099473; doi:10.1111/evo.13496)
Supplement: Supplementary file 1 — Table S1. Pedigree restrictions on number of known grandparents affected not only the sample size, but also the rates of incestuous pairings between social partners (kSOC > 0) and inbreeding (f > 0). Table S2. Effects of kinship between the social male and female and the effects of helpers on (a) clutch size, and (b) hatchability/survival of offspring to measurement age. Output from MCMCglmm models: sample sizes are number of broods across 26 cohorts. Table S3. Test for inbreeding depression and the effects of social environment on the magnitude of inbreeding depression (an interaction between the inbreeding coefficient and number of helpers). Table S4. Effects of kinship between the social male and female and the effects of helpers on the proportion of extra‐pair offspring in the brood, including an interaction between kSOC and helpers. Table S5. Effects of pedigree restrictions on numbers and percentages of inbred and outbred within‐ and extra‐pair offspring. Table S6. Pedigree restrictions: effects of within‐pair status of an individual (whether it was within‐pair, WP, or extra‐pair, EP) on the individual's inbreeding status (whether it was inbred, with f > 0, or outbred, with f = 0), using binomial mixed models run in MCMCglmm. Table S7. Pedigree restrictions: effects of within‐pair status of an individual (whether it was within‐pair, WP, or extra‐pair, EP) on the individual's inbreeding status (whether it was inbred, with f ≥ 0.0625, or outbred, with f < 0.0625), using binomial mixed models run in MCMCglmm. Table S8. Effects of social environment ‐ number of helpers – on the probability of offspring being inbred. Table S9. Predicted nestling mass (g) for increasing levels of inbreeding (standard errors in brackets). [file EVO-72-1500-s001.docx]

**SUPPLEMENTARY MATERIAL**

Table of Contents

[SUMMARY OF SI RESULTS 2](#_Toc509245898)

[SI METHODS 3](#_Toc509245899)

[**Methods - Paternity assignment** 3](#_Toc509245900)

[**Methods SECTION 1: Pedigree reconstruction and quantifying levels of inbreeding** 7](#_Toc509245901)

[**Methods SECTION 2: Inbreeding depression** 8](#_Toc509245902)

[**Methods SECTION 3: Effects of kinship between social partners on infidelity** 9](#_Toc509245903)

[**Methods SECTION 4: Effects of infidelity on inbreeding** 10](#_Toc509245904)

[**FIGURES FITTING** 11](#_Toc509245905)

[SI FURTHER ANALYSES 11](#_Toc509245906)

[**SECTION 1** 11](#_Toc509245907)

[**Pedigree restrictions: effects of grandparents’ restrictions** 11](#_Toc509245908)

[**SECTION 2** 13](#_Toc509245909)

[**Inbreeding depression in early survival. Does kinship of social partners affect clutch size or survival to measurement?** 13](#_Toc509245910)

[**Effect of social environment on inbreeding depression** 15](#_Toc509245911)

[**SECTION 3** 18](#_Toc509245912)

[**Does kinship of social partners affect clutch size or survival to measurement?** 18](#_Toc509245913)

[**Effects of social environment on association between k_SOC_ and infidelity** 18](#_Toc509245914)

[**SECTION 4** 19](#_Toc509245915)

[**Pedigree restrictions: effects of infidelity on the probability of offspring being inbred** 19](#_Toc509245916)

[**MCMCglmm truncation of latent variables** 20](#_Toc509245917)

[**Effects of pedigree restrictions on the probability of offspring being inbred** 21](#_Toc509245918)

[**Classification by f ≥ 0.0625 rather than f > 0** 22](#_Toc509245919)

[**Effects of social environment on the probability of offspring being inbred** 23](#_Toc509245920)

[**SECTION 5: Additional analyses following review** 25](#_Toc509245921)

[REFERENCE LIST 28](#_Toc509245922)

# SUMMARY OF SI RESULTS

**Section 1:** Pedigree restriction had a considerable effect on the estimates of inbreeding, *k_SOC_*, and the sample sizes (Table S1). Most notably, inbreeding rates increased sharply once 3+ known grandparents’ restriction was applied. **Section 2:** There was no indication of early inbreeding depression (Table S2). The social environment did not mitigate the effects of inbreeding depression on nestling mass or survival (Table S3). **Section 3:** Neither the relatedness between social partners (*k_SOC_)* nor the number of helpers had any effect on the clutch size and/or survival to measurement age (Table S2). The association between *k_SOC_* and infidelity was not affected by the social environment (Table S4). **Section 4:** We show that our results of within-pair offspring being more likely to be inbred than extra-pair offspring are consistent across (a) pedigree restrictions (Table S6); (b) classification of “inbred” individuals (Table S7). Furthermore, social environment was not associated with the probability of offspring being inbred (Table S8).

# SI METHODS

## **Methods - Paternity assignment**

The methods we use to resolve paternity in the superb fairy-wren study population were described in Double et al. (1997b) and used subsequently for analyses of mating patterns and reproductive success in our study population; see for example Cockburn et al. (2003); Double & Cockburn (2003); and Cockburn et al. (2008). In brief, the system was based on microsatellite genotyping and involved eight polymorphic microsatellite loci (McyU1-McyU8). Usually in paternity assignment, the probability of excluding a falsely assigned father is calculated in order to assess the utility of the loci. However, this exclusion probability does not account for the population structure present in superb fairy-wrens (clustering of relatives due to male philopatry, presence of reproductively-active male helpers, and male-female reproductive success asymmetry). Because of this, Monte Carlo simulation models that use allele frequencies for multiple loci to estimate the exclusion probabilities when multiple male first-order relatives are considered to be potential sires were developed (Double et al. 1997a). These models suggested that standard exclusion probability approach was in fact not accurate enough for use in the superb fairy-wren system due to the natal male philopatry and resulting loss of resolution when multiple male relatives are competing for paternity. Therefore, more realistic exclusion probabilities can be obtained by taking into account the characteristics of the system: in this case by assuming that there are close relatives amongst the potential sires and then deciding on the number of loci required to estimate robust paternities, as outlined below.

Subsequent to the initial development of the methods described by Double et al. (1997b), we encountered a deterioration in the utility of some of the microsatellite loci, probably because of the introduction of null alleles. We therefore replaced three loci, discarding McyU1, McyU5 and McyU6 and replacing them with one marker developed for the congeneric *Malurus splendens* (MspU19, Webster et al. (2004)), one from the confamilial *Stipiturus malachurus* (SmmU7, Maguire et al. (2006)), and the third from *Terpsiphone mutata* (TmmU6, Adcock & Mulder (2002)). The first two of these are moderately variable (approx. 10 alleles in our population), but TmmU6 is hypervariable, with more than 50 alleles.

Our method does not rely on the available statistical programs to ascribe paternity, as the primary problem where ambiguity arises reflects competition between close male relatives such as fathers and sons, or full siblings (Double et al. 1997a), and we also need to allow for moderate mutation rates in key loci (Beck et al. 2003). We therefore assigned paternity following a series of steps developed specifically to handle these scenarios.

We initially compared young with their mother to identify which alleles in the offspring could have had a paternal origin. Maternity is always known as there is no evidence of egg-dumping in this species. We then examined the social partner of the female to assess his suitability as a sire. Regardless of a match with the social partner, we then tested the ability of all the males known to be alive when the female was fertile to sire the offspring as well. We also included a further set of males defined as ‘potentially alive’ in this candidate pool, which were birds that have been caught intruding into the study area on courtship displays, and whose territory was not known or monitored so that death date could be precisely determined. We included these extra males in all the years that they might have reasonably been alive: we did not allow the potential sires to exceed 12 years of age, which is the maximum recorded age in the study population.

In the first stage we defined a match at a locus as being within one repeat unit within the microsatellite (2 and 4 bases respectively for dinucleotide and tetranucleotide loci) of the offspring allele or alleles, as most mutations involve a single addition or deletion of a repeat unit (Beck et al. 2003).

In some cases the social partner of the female at the time she had a nest was never caught, and we did not attempt to attribute parentage in these cases, as the male could not be reliably excluded. Subsequent to the study area reaching its full size in 1993 we recorded 10 such offspring in nests attended by 3 different social partners. Just two of these offspring went on to produce young themselves, so the effect of not attributing paternity in these cases on the integrity of the pedigree is limited.

Although in general we unambiguously identified a single best sire, we encountered a number of ambiguities that we resolved with either further analysis or pragmatic rules-of-thumb.

Most commonly (*n* = 654 offspring), we found that all the males in the comparison set could be reliably excluded as sires (generally < 75% matches with the offspring’s paternally inherited alleles). There is very strong evidence that these offspring were sired by males living outside the study area. First, they were much more prevalent in territories close to the boundary of the study area. Second, the high success of some of our ‘potentially alive’ class attests to the importance of out-of-area males: the most successful of these produced 13 and 11 young and 51 and 37 grand-offspring respectively. For the pedigree and calculation of inbreeding coefficients, ignoring the possibility that the offspring had the same out-of-area sire can potentially lead to full sibs being treated as half sibs. It is most parsimonious but by no means inevitable that the offspring in these cases are likely to have just one extra-group sire. We used the program COLONY (Jones & Wang 2010) to identity offspring that were likely to be sired by the same out-of-area male, which is the parsimonious conclusion as clutches are likely to have just one extra-group sire. We could not examine between-year patterns in a single analysis because of computational limitations. We did examine young within subregions of the study area to see whether the same male had been successful in consecutive years. This has thus far led us to recognise 108 unknown males which we treated as sires in the pedigree, which contributed 328 young (50.2% of the original 654). We should note that the COLONY test is probably conservative when the number of offspring is just two.

The next problem was the identification of two (or very rarely more) males that matched the young equally according to our initial criteria. These were generally father-son or full-sib pairs. We distinguished between the possibilities according to the following criteria, which are arranged in descending order of frequency. If the matching of one male depended on allowing for mutations but the other did not, we assigned to the male that did not require the assumption of mutation. Second, if one of the competing males exclusively matched some of the other young in the brood or in consecutive broods by the same female, but the second had no unique matches, we assigned paternity to the male with exclusive matches. Third, in a number of cases a male socially paired to his mother matched the offspring, but so did his own extra-group sire (*n* = 5 offspring from 5 broods by five different mother-offspring pairings). We have strong behavioural evidence that males show no sexual interest in their mothers, so in this case we assigned paternity to extra-group sire. There were also 3 offspring where a son that had been sired by an out-of-area male appeared to have been successful with his mother to which he was socially paired. Because this only occurred in other cases within the study area when the son’s sire was alive, we assumed that this was also true in these cases, and we assessed these young to have been produced by extra-group parentage. Finally, for just 3 young we got a match that lived a very great distance from the mother (greater than 6 territories), and a match with a male that lived nearby. In these cases we assigned paternity to the nearby male.

There were 72 offspring that could not easily be distinguished according to these criteria, so we did not assign parentage. Fortunately, 69 of these (96%) produced no young themselves, and hence had no influence on the subsequent structure of the pedigree.

## **Methods SECTION 1: Pedigree reconstruction and quantifying levels of inbreeding**

Using the parentage data, we constructed a pedigree containing individuals sampled between 1988 and 2013. The maximum lineage length in the pedigree was 15 generations. Individuals without known parents were assumed to be unrelated to the rest of the population. Detecting inbreeding requires a minimum knowledge of identity of both parents and at least one grandparent; we therefore only estimated inbreeding coefficients (*f*) for individuals where the identities of both genetic parents and at least one grandparent were known (*n* = 4431). The depth and accuracy of pedigree data can affect estimates of the prevalence of inbreeding, but restricting a pedigree to individuals with larger amounts of ancestry information inevitably comes at a cost to sample sizes (Walling et al. 2011). We provide details of the effect of restrictions on the sample sizes and inbreeding rates (Table S1).

For all social pairs (*i.e.* the dominant male, who was always the oldest male on the territory, and the breeding female), we calculated a kinship coefficient (*k_SOC_*), defined as the probability that two homologous alleles, one sampled at random from each individual, are identical by descent (Wright 1922). Similarly, we calculated a kinship coefficient (*k_EP_*) for each female-EP male pair that produced extra-pair offspring. For each offspring, we calculated an inbreeding coefficient (*f*), defined as the probability that two alleles at any randomly-chosen locus in the individual are identical by descent (Wright 1922). Both the pedigree reconstruction and the *k* and *f* calculations were carried out using the *pedigreemm* R package (Vazquez et al. 2010). Throughout, we distinguish between the ‘genetic father’, to mean the male who sired a particular offspring, and the ‘social father’, to mean the male who was dominant on the territory at the time that the offspring was hatched, and who may or may not have been the genetic father.

Our main dataset therefore contained 4431 individuals with known genetic parents and at least one known grandparent. These came from 1745 broods, involving 579 mothers and 615 genetic fathers, with 1726 unique pairings between mothers and fathers, out of which 1197 involved EP fathers. The 1745 broods came from 863 unique social pairings, involving 579 mothers and 536 social fathers.

The variables fitted in the statistical models varied depending on the model, thus sample sizes for individual models varied and are given alongside the model results. All analyses were carried out in *R* version 3.3.1 (Development Core Team 2011).

## **Methods SECTION 2: Inbreeding depression**

**Inbreeding depression on survival from fledging to independence (41 days)**.

Since we were interested in individual-level effects of inbreeding on survival, post-fledging survival is more relevant than survival at the nestling stage, where mortality is largely due to predation of the entire brood. We therefore tested for inbreeding depression in survival to independence. Fledging in our study population usually happens at 12 days and the upper bound of 41 days was chosen as an approximate measure of independence: some young receive provisioning at this age, but the earliest known dispersal of young in our study happened at 41 days post-fledging, showing that the individuals can be independent after that point. During the breeding season we aim to census each bird at least three times each week (Cockburn et al. 2003), thus, “death date” for each individual can be estimated with reasonable accuracy. The effects of survival across other life-history stages (up to recruitment into the breeding population) were also investigated, but since those models gave comparable results they are not presented here.

**Model details:** To test for inbreeding depression on survival from fledging to 41 days, we ran two generalised linear mixed effects animal models using the *MCMCglmm* package (Hadfield 2010). We first tested whether inbreeding affected survival, and then investigated whether any effect of inbreeding acted through body mass, by including mass as a covariate in the analysis to check whether the inbreeding coefficient changed. Survival was modelled as a binary (0/1) response variable, with a binomial error distribution. **Fixed Effects:** *Inbreeding coefficient (f)*, the *number of helpers*, *brood size* and an *individual’s sex* were fitted as described above (with *nestling mass* as an additional covariate in one of the models). **Random Effects:** *Nest ID, additive genetic effect, cohort* and *hatch date* were fitted, as described above.

We ran the analyses for 3.9x10^6^ iterations with a burn-in of 9x10^5^ and a thinning interval of 1500, generating 2000 samples from which posterior means and 95% CIs (credible intervals; lower CI, upper CI) were calculated. We used parameter expanded priors (*V* = 1, *fix* = 1, *nu* = 1, *alpha.mu* = 0, *alpha.V* = 1x1000), following the documentation available for the *MCMCglmm* package, such as the *MCMCglmm* *Course Notes* by Jarrod Hadfield (see *MCMCglmm*’s CRAN page: <https://cran.r-project.org/web/packages/MCMCglmm/vignettes/CourseNotes.pdf>).

**All *MCMCglmm* models:** In all models we ensured that these effective sample sizes were above 1000, as effective sample sizes for specific parameters could be lower than 2000 due to presence of autocorrelation. Additionally, any missing values were removed from the fixed predictors in all models, i.e. there were no birds of unknown sex or age used in any of the models. Note that the removal of missing values from fixed predictors affected sample sizes used by specific models. No stepwise model selection was used: all fitted parameters were chosen based on their (potential) biological significance.

## **Methods SECTION 3: Effects of kinship between social partners on infidelity**

We fitted binomial generalised linear mixed models using the R package *MCMCglmm,* with the proportion of extra-pair offspring in a brood (defined by the numbers of extra- *vs.* within-pair offspring) as a response variable, and binomial errors; these models were by definition fitted at the level of the brood rather than individual nestlings. **Fixed Effects:** *Kinship* (continuous) was fitted to test whether the probability of offspring being sired by an extra-pair male varied with the kinship between the female and her social mate (*k_SOC_*). The *number of helpers* (0, 1 and 2+) was fitted to test whether the social environment affected the probability of extra-pair reproduction. Additionally, the *mother’s age* and the *social father’s age* (two level factors: one year old, older) were fitted as fixed effects to account for potential effects of differences in experience. **Random Effects:** *Mother ID* and *social father ID* were fitted to account for the multiple observations on specific females and males (social fathers). We also fitted a multi-level factor of *cohort* to account for inter-annual variation.

**Model details:** *iterations* = 2.6x10^6^; *burn-in* = 6x10^5^; *thinning interval* = 1000; generating 2000 samples; with parameter expanded priors: *V* = 1, *n* = 0.002, *nu* = 1, *alpha.mu* = 0, *alpha.V* = 1x1000.

## **Methods SECTION 4: Effects of infidelity on inbreeding**

**Step 1.** We fitted a binomial generalised linear mixed model using *MCMCglmm*, with the inbreeding status of every offspring as a response (two level factor: inbred *vs*. outbred, where an inbred individual was defined as one for which *f* > 0). **Fixed Effects:** We fitted *within-pair status* (whether an offspring was the result of within-pair (WP) or extra-pair (EP) reproduction) as a two-level factor (WP, EP). **Random Effects:** *Nest ID* was fitted to account for similarities across multiple offspring from the same brood. We also fitted a multi-level factor of *cohort* to represent inter-annual variation. The model was fitted excluding mother-son pairings to avoid any bias stemming from those special cases.

**Step 1 model details:** *iterations* = 3.9x10^6^; *burn-in* = 9x10^5^; *thinning interval* = 1250; generating 2400 samples; with parameter expanded priors: *V* = 1, *fix* = 1, *nu* = 1, *alpha.mu* = 0, *alpha.V* = 1x1000.

**Step 2.** We then tested what determined the magnitude of the inbreeding coefficient amongst those nestlings that were inbred, i.e. had *f* > 0. We fitted a linear mixed model using the *MCMCglmm* package, with log-transformed inbreeding coefficient (*f*) as the response variable, and Gaussian errors. We used only the inbred individuals (*f* > 0) in this model. **Fixed Effects:** We fitted *within-pair status* (whether an offspring was the result of within-pair (WP) or extra-pair (EP) reproduction) as a two-level factor (WP, EP). **Random Effects:** As before, *nest ID* and *cohort* were fitted as random effects. The model was fitted excluding mother-son pairings to avoid any bias stemming from those special cases.

**Step 2 model details:** *iterations* = 2.6x10^6^; *burn-in* = 6x10^5^; *thinning interval* = 1000; generating 2000 samples; with parameter expanded priors: *V* = 1, *n* = 0.002, *nu* = 1, *alpha.mu* = 0, *alpha.V* = 1x1000.

## **FIGURES FITTING**

Figures were plotted in R using the *ggplot2* package (Wickham 2009). Predictions in Figure 1a and Figure 2 are based on GLMMs fitted in *lme4* (Bates et al. 2015) models equivalent to the *MCMCglmm* models presented in the main text (without the additive genetic effect for Figure 1a). This is because we encountered difficulties in obtaining and plotting predictions from *ASReml-R* (Butler et al. 2009) and *MCMCglmm* packages used to run the main models.

# SI FURTHER ANALYSES

## **SECTION 1**

### **Pedigree restrictions: effects of grandparents’ restrictions**

Since the depth and accuracy of the pedigree data can affect estimates of inbreeding, we restricted our dataset accordingly to investigate the impact of the different levels of ancestry information on samples sizes and estimates of the frequency of inbreeding. Our main dataset included all individuals with known genetic parents and at least one known grandparent, while further restrictions focused on the number of known grandparents: 2+, 3+ and all 4 grandparents known.

Pedigree restrictions had a considerable effect on the estimates of inbreeding in our population, with increasing level of restrictions resulting in increasing rates of incestuous pairings and of inbreeding (Table S1). A restriction of requiring 4 grandparents reduced the data sets to 22.7% (broods) and 22.1% (individuals) of their original sizes, but increased the percentage of *k_SOC_* > 0 and *f* > 0 by 18% and 16% respectively. The increased frequency presumably reflects in part the exclusion of pairs/individuals erroneously assigned *k_SOC_* = 0 and *f* = 0 because their ancestry information was not sufficient to identify lower levels of relatedness/inbreeding. However, it is worth noting that the marked increase in inbreeding rate beyond 2+ known grandparents is most likely due to restricting the dataset towards females who have dispersed shorter distances from their natal territory, as our ability to sample all grandparents was often conditioned on these females, and they may be more likely to encounter male relatives as partners than females that disperse over longer distances. In an analysis of a multigenerational red deer pedigree (Walling et al. 2011), similar reductions in sample size and increases in inbreeding with stricter restrictions were notes, but with a relatively consistent change with increasing restrictions (J. Huisman pers. comm.), rather than the sharp jump observed here from 2+ to 3+ known grandparents.

Table S1. Pedigree restrictions on number of known grandparents affected not only the sample size, but also the rates of incestuous pairings between social partners (k_SOC_ > 0) and inbreeding (f > 0).

## **SECTION 2**

### **Inbreeding depression in early survival. Does kinship of social partners affect clutch size or survival to measurement?**

Note that this analysis is important for both Section 2, as it gave us an indirect test of inbreeding depression in the wild, and for Section 3, as it allowed us to ensure that our estimates of extra-pair offspring in the brood were not biased. (Note: in response to reviewer comments we also ran several additional analyses, which are summarised in Section 5 below).

It is possible that inbreeding depression in very early survival could bias later estimates of the extent of extra-pair paternity in pairs where social partners are related: in short, if all within-pair offspring died due to inbreeding depression, the brood would appear to be composed of entirely extra-pair offspring and associations of EPP with relatedness of social partners will be overestimated (Reid 2015; Reid et al. 2015). In addition, there is the possibility that females may chose to lay smaller clutches when socially paired to related males (Duthie et al. 2016). To assess the likelihood of these scenarios affecting our estimates, we first tested whether kinship between the social partners affected clutch size and/or survival of nestlings to measurement age (survival to measurement age covers both hatchability and subsequent survival of hatched young). We defined clutch size as the number of eggs initially laid, and survival to measurement as the ratio of brood size (the number of offspring in a nest at banding age) to clutch size.

We fitted generalised linear mixed effects models in the *MCMCglmm* package. We fitted (a) clutch size, with Gaussian errors, (b) the proportion of nestlings making it to measurement age (brood size / clutch size) with binomial errors, as responses. **Fixed Effects:** A fixed effect of kinship (continuous) was fitted to investigate whether either brood or clutch size decreased with *k_SOC_*. The number of helpers (0, 1 and 2+) was also fitted to test whether the social environment affected egg/nestling production. Additionally, mother’s age and social father’s age (two level factors: one-year-old, older) were fitted as fixed effects to account for potential differences in experience and/or investment with age. **Random Effects:** Mother ID and social father ID were fitted as random effects to account for the multiple observations on specific females and males (social fathers). We also fitted a multi-level factor of cohort to represent inter-annual variation.

We run the analyses for 5.2x10^5^ iterations with a burn-in of 1.2x10^5^ and a thinning interval of 200, generating 2000 samples from which posterior means and 95% CIs (lower CI, upper CI) were calculated. For all *MCMCglmm* models the effective sample sizes for specific parameters varied due to autocorrelation, but we ensured that they were all above 1000. Similarly, for all *MCMCglmm* models we considered terms to be statistically significant based on 95% CIs not spanning 0 and *pMCMC* values (number of simulations greater or smaller than 0 corrected for number of *MCMC* samples) calculated by *MCMCglmm* being < 0.05. We run models with parameter expanded priors (*V* = 1, *n* = 0.002, *nu* = 1, *alpha.mu* = 0, *alpha.V* = 1x1000).

**Results:** Neither the relatedness between social partners (*k_SOC_)* nor the number of helpers had any effect on the clutch size and/or survival to measurement age (Table *S2*). This analysis therefore provided an additional indirect test of inbreeding depression in early survival, and gave no indication of early inbreeding depression.

Table S2. Effects of kinship between the social male and female and the effects of helpers on (a) clutch size, and (b) hatchability/survival of offspring to measurement age. Output from MCMCglmm models: sample sizes are number of broods across 26 cohorts.

### **Effect of social environment on inbreeding depression**

We then tested for inbreeding depression in nestling mass and survival (see the main text for details). Here we present the inbreeding depression models fitted with an interaction between the inbreeding coefficient (f) and the number of helpers, to test for any effects of social environment and assess whether the presence of helpers could mitigate any effects of inbreeding depression.

To test for inbreeding depression in nestling mass, we fitted a linear mixed effects animal model fitted using the ASReml-R package, with nestling mass as the response, with Gaussian errors. **Fixed Effects:** inbreeding coefficient (f), number of helpers, brood size, sex of nestling, nestling age at measurement (quadratic function). We also fitted a two level factor (pre-1992, 1992+) to account for the introduction of a new weighing protocol in 1992 (Kruuk et al. 2015); and an interaction between the inbreeding coefficient and the number of helpers. **Random Effects:** nest ID, an additive genetic effect (with covariance structure determined by the pedigree), cohort, hatch date.

To test for inbreeding depression in survival from fledging to 41 days, we fitted generalised linear mixed effects animal models fitted using the MCMCglmm package. We ran one model with nestling mass fitted as a covariate, as mass may affect survival, and a second model without nestling mass; with survival as a binary (0/1) response variable, and a binomial error function. Iterations = 3.9x10^6^, burn-in = 9x10^5^, thinning interval = 1500, samples generated = 2000. Parameter expanded priors: V = 1, fix = 1, nu = 1, alpha.mu = 0, alpha.V = 1x1000. **Fixed Effects:** inbreeding coefficient (f), number of helpers, brood size and offspring sex (with mass as a covariate in the first model). We also fitted an interaction between the inbreeding coefficient and the number of helpers. **Random Effects:** nest ID, additive genetic effect, cohort and hatch date.

**Results:** The social environment did not mitigate the effects of inbreeding depression on nestling mass or survival (Table S3).

Table S3. Test for inbreeding depression and the effects of social environment on the magnitude of inbreeding depression (an interaction between the inbreeding coefficient and number of helpers). Effects of inbreeding coefficient f on **(a)** nestling mass; and on survival from fledging to 41 days fitted **(b)** with nestling mass (corrected for change in protocol in 1992 and for nestling age at measurement) included as a covariate, and **(c)** without nestling mass included. (Note that the precise form of output differs for the ASReml-R model in (a) versus the MCMCglmm models in (b)/(c).)

## **SECTION 3**

### **Does kinship of social partners affect clutch size or survival to measurement?**

Details of the analysis are presented in the above section, as this analysis provided us with an indirect text of inbreeding depression in early survival.

**Results:** Neither the relatedness between social partners (*k_SOC_)* nor the number of helpers had any effect on the clutch size and/or survival to measurement age (Table S2).

### **Effects of social environment on association between *k_SOC_* and infidelity**

We tested whether patterns of extra-pair paternity were associated with either kinship between social partners (*k_SOC_*) and/or social environment, specifically the presence of helpers at the nest. Two models were run: (a) using all available data, including the mother-son pairings; and (b) excluding the mother-son pairings. See the main paper for details. Here we present these models fitted with an interaction between *k_SOC_* and the number of helpers, to test for the role of social environment.

We fitted a binomial generalised linear mixed model using the *MCMCglmm* package, with the proportion of extra-pair offspring in a brood (using the numbers of extra- and within-pair offspring) as a response variable, with binomial errors (*iterations* = 2.6x10^6^; *burn-in* = 6x10^5^; *thinning interval* = 1000; generating 2000 samples; with parameter expanded priors: *V* = 1, *n* = 0.002, *nu* = 1, *alpha.mu* = 0, *alpha.V* = 1x1000). **Fixed Effects:** kinship (*k_SOC_*), number of helpers, mother’s age and social father’s age. Interaction between *k_SOC_* and the number of helpers. **Random Effects:** mother ID, social father ID, cohort.

**Results:** There was no significant interaction between *k_SOC_* and social environment (Table S4).

Table S4. Effects of kinship between the social male and female and the effects of helpers on the proportion of extra-pair offspring in the brood, including an interaction between k_SOC_ and helpers. Models were run **(a)** on all data, including mother-son pairings; and **(b)** excluding mother-son pairings and any offspring produced by females socially paired to their sons

## **SECTION 4**

### **Pedigree restrictions: effects of infidelity on the probability of offspring being inbred**

We showed above that pedigree restrictions had a substantial effect on inbreeding rates (Table S1). For completeness, we also present the effects of pedigree restrictions on the numbers and percentages of inbred and outbred within- and extra-pair offspring (Table S5).

Table S5. Effects of pedigree restrictions on numbers and percentages of inbred and outbred within- and extra-pair offspring. Percentages are presented per row and rounded to 1 decimal place. Any individual with inbreeding coefficient f > 0 was classified as inbred.

Further, we ran models testing the consequences of extra-pair paternity for the probability of offspring being inbred imposing the above pedigree restrictions, to ensure that the analyses in Table 5 were robust to these restrictions.

### ***MCMCglmm* truncation of latent variables**

Note that all models in this section, regardless of restrictions, were run with an adjusted version of the *MCMCglmm* package allowing for truncation of latent variables: any latent variable that is less than -25 or greater than 25 was rejected at the Metropolis-Hastings stage to avoid under/overflow. This was necessary due to considerable variation in mortality between nests: in most cases either the nest fledged fully or completely failed, and thus there was little partial mortality, which lead to problems with extreme latent variables. We thank Jarrod Hadfield for providing the adjusted version of *MCMCglmm*.

### **Effects of pedigree restrictions on the probability of offspring being inbred**

We fitted a binomial generalised linear mixed models using the *MCMCglmm* package, with the inbreeding status of every offspring as a response (two level factor: inbred *vs*. outbred, where an inbred individual was defined as one for which *f* > 0). **Fixed Effects:** within-pair status (whether an offspring was the result of within-pair (WP) or extra-pair (EP) reproduction). **Random Effects:** nest ID, cohort. The models were fitted excluding mother-son pairings to avoid any bias stemming from those special cases. Additionally, because the *number of helpers* affects rates of extra-pair paternity, we did not fit the number of helpers in these models, given the potential confounding effects between the two.

Model settings. **For** **1+ known grandparent**: *iterations* = 3.9x10^6^; *burn-in* = 9x10^5^; *thinning interval* = 1250; generating 2400 samples; **for 2+ known grandparents**: *iterations* = 5.2x10^6^; *burn-in* = 1.2x10^6^; *thinning interval* = 2000; generating 2000 samples; **for** **3+ and 4 known grandparents**: *iterations* = 2.6x10^6^; *burn-in* = 0.6x10^6^; *thinning interval* = 1000; generating 2000 samples. We used parameter expanded priors: *V* = 1, *nu* = 1, *alpha.mu* = 0, *alpha.V* = 1x1000.

**Results:** Results were consistent across models regardless of the restriction applied: within-pair offspring were more likely to be inbred than extra-pair offspring, even though mother-son pairings were excluded from the datasets (Table S6).

Table S6. Pedigree restrictions: effects of within-pair status of an individual (whether it was within-pair, WP, or extra-pair, EP) on the individual’s inbreeding status (whether it was inbred, with f > 0, or outbred, with f = 0), using binomial mixed models run in MCMCglmm. The models were run without mother-son pairings.

### **Classification by *f* ≥ 0.0625 rather than *f* > 0**

We treated every individual with inbreeding coefficient *f* > 0 as inbred in the above analyses and in the analyses presented in the main text. However, the large majority of inbred individuals (94.3%) had inbreeding coefficients *f* < 0.125. Therefore, in order to ensure that the many low inbreeding values were not affecting the results disproportionately, we also ran the models on the effects of infidelity on the probability of offspring being inbred using *f* = 0.0625 as a cut-off point: i.e. only individuals with *f* ≥ 0.0625 were treated as inbred. As above, we also checked whether restrictions based on different numbers of grandparents affected the results and we did not fit the number of helpers in these models, given the potential confounding effects between the two.

We fitted a binomial generalised linear mixed model using *MCMCglmm*, with the inbreeding status of every offspring as a response (inbred *vs*. outbred, where an inbred individual was defined as one for which *f* ≥ 0.0625). **Fixed Effects:** within-pair status (WP *vs.* EP). **Random Effects:** nest ID, cohort. The model was fitted excluding mother-son pairings to avoid any bias stemming from those special cases.

Model settings. For **1+ known grandparent**: *iterations* = 3.9x10^6^; *burn-in* = 9x10^5^; *thinning interval* = 1250; generating 2400 samples; for **2+ known grandparents:** *iterations* = 5.2x10^6^; *burn-in* = 1.2x10^6^; *thinning interval* = 2000; generating 2000 samples; for **3+ and 4 known grandparents**: *iterations* = 2.6x10^6^; *burn-in* = 0.6x10^6^; *thinning interval* = 1000; generating 2000 samples. We used parameter expanded priors: *V* = 1, *nu* = 1, *alpha.mu* = 0, *alpha.V* = 1x1000.

**Results:** Table S7 show that the results were nearly identical (compare to Table S6) whether inbred individuals were classified as *f*> 0 or *f* ≥ 0625, regardless of grandparent restriction applied.

Table S7. Pedigree restrictions: effects of within-pair status of an individual (whether it was within-pair, WP, or extra-pair, EP) on the individual’s inbreeding status (whether it was inbred, with f ≥ 0.0625, or outbred, with f < 0.0625), using binomial mixed models run in MCMCglmm. The models were run without mother-son pairings.

###

### **Effects of social environment on the probability of offspring being inbred**

Finally, we also fitted models looking at the probability of offspring being inbred as a response with the number of helpers as a sole fixed effect in order to test for the influence of the social environment. We fitted two binomial generalised linear mixed models using *MCMCglmm* two models: (a) inbred *f* > 0 and (b) inbred *f* ≥ 0.0625. Inbreeding status of every offspring was fitted as a response (inbred *vs*. outbred). **Fixed Effects:** number of helpers. **Random Effects:** nest ID, cohort. The models were fitted excluding mother-son pairings to avoid any bias stemming from those special cases.

Model settings: (a) *iterations* = 2.6x10^6^; *burn-in* = 0.6x10^6^; *thinning interval* = 1000; generating 2000 samples; (b) *iterations* = 3.9x10^6^; *burn-in* = 9x10^5^; *thinning interval* = 1250; generating 2400 samples. We used parameter expanded priors: *V* = 1, *nu* = 1, *alpha.mu* = 0, *alpha.V* = 1x1000.

**Results:** Helpers did not have an effect on the offspring’s probability of being inbred (Table S8).

Table S8. Effects of social environment - number of helpers – on the probability of offspring being inbred. Two different ways of classifying “inbred” individuals were used (a) inbred were f > 0, (b) inbred were f ≥ 0/0625. These binomial generalized linear mixed models were run in MCMCglmm.

## **SECTION 5: Additional analyses following review**

In response to reviewer requests we ran several additional analyses. These involved:

1. A test of whether **inbred offspring are differentially cared for**, which could offset the negative effects of such inbred offspring having lower nestling mass, thus explaining no evidence of inbreeding depression in fledgling survival to independence despite reduced survival of lighter offspring. We assumed that parents cannot distinguish between inbred and outbred nestlings within a brood and therefore all offspring in a brood including inbred nestlings would have to receive increased parental care. If true, outbred offspring raised in the nest with inbred offspring should have higher mass than outbred offspring in ‘fully-outbred’ nests. We ran a model in *ASReml-R*, with the nestling mass of outbred offspring as a response (Gaussian errors) and ‘sharing the nest with inbred offspring’ (yes/no) as a fixed effect. Other fixed and random effects fitted followed the model in Table 2a (but of course without fitting the inbreeding coefficient, as all used offspring were outbred). **Results:** We found no effect of sharing the nest with inbred offspring on outbred nestlings’ mass (*pMCMC* = 0.623), indicating that parents do not adjust their investment in the presence of inbred offspring.
2. A test of whether **the** **inbreeding coefficient of offspring is affected by kinship** between the social partners as the relationship between clutch mortality (selective disappearance) and kinship(*k_SOC_*) could be affected if *k_SOC_* causes a female to differentially mate with extra-pair males of specific relatedness. We ran the model in *MCMCglmm*, with Gaussian errors, log-transformed response, and parameter expanded priors. The traces, autocorrelation, and effective sample sizes were satisfactory, although posterior means appeared rather high. **Results:** Kinship was not associated with the inbreeding coefficient values of offspring (*pMCMC* = 0.958).
3. A test of whether **the number of extra-pair sires per brood increased with kinship** between the social partners, as the relationship between clutch mortality (selective disappearance) and *k_SOC_* could be affected if *k_SOC_* causes a female to change the number of extra-pair sires per clutch. We ran a model in *MCMCglmm*, with Poisson errors, the number of extra=pair sires per brood as a response, the kinship as the sole fixed effect, and the mother ID, social father ID and cohort as random effects. We ran the model both with and without the mother-son pairings. **Results:** The number of extra-pair sires in a brood (1, 2, 3 or 4 – only one case of 4) was not associated with kinship between the social partners (*pMCMC* = 0.883).
4. Opportunity cost. Reviewer wondered whether the lack of mother-son mating between mother-son social pairs is linked to such matings affecting the son’s opportunity to mate with other females (extra-pair). We compared the EP success of dominants in mother-son pairs and other dominants. **Results:** There was no evidence that being paired to mothers affected male’s EP success compared to other dominant males (Chi-squared test, *p* = 0.324).
5. The reviewer also enquired about the effects of grandparents’ restrictions on the inbreeding depression and the severity of inbreeding depression. We provide an additional table (see below) showing predicted nestling mass for different grandparent restrictions, across a range of inbreeding coefficients. It clearly shows that the magnitude of the inbreeding depression in mass does not change dramatically across the restrictions, at the same time drawing attention to 1+/2+ and 3+/4 restrictions encompassing a different set of individuals (Table S9), which, together with the sample-size trade-off, was part of the reason we decided to present results for the ‘at least one grandparent’ restriction.

Table S9. Predicted nestling mass (g) for increasing levels of inbreeding (standard errors in brackets). Predictions were obtained from the *ASReml-R* models by averaging the fixed effects with the predict.asreml function.

|  | restriction level - number of known grandparents | | | |
| --- | --- | --- | --- | --- |
| inbreeding coefficient (*f*) | 1+ | 2+ | 3+ | 4 |
| 0 | 7.26 (0.07) | 7.25 (0.08) | 7.13 (0.20) | 6.87 (0.24) |
| 0.0156 | 7.20 (0.08) | 7.20 (0.08) | 7.08 (0.20) | 6.83 (0.24) |
| 0.03125 | 7.15 (0.08) | 7.14 (0.09) | 7.03 (0.20) | 6.78 (0.24) |
| 0.0625 | 7.03 (0.11) | 7.03 (0.11) | 6.93 (0.22) | 6.69 (0.26) |
| 0.125 | 6.81 (0.17) | 6.81 (0.18) | 6.74 (0.28) | 6.51 (0.32) |
| 0.25 | 6.35 (0.32) | 6.37 (0.34) | 6.36 (0.44) | 6.15 (0.48) |
| % difference (mass for *f­* = 0 *vs. f* = 0.25) | 12.5% | 12.1% | 10.8% | 10.5% |
| total sample size per restriction | 4167 | 3805 | 1222 | 922 |

# REFERENCE LIST

Adcock G.J. & Mulder R.A. 2002. Polymorphic microsatellite loci for paternity analysis in the Madagascar paradise flycatcher (*Terpsiphone mutata*: Aves). *Molecular Ecology Resources* 2: 287-289.

Bates D., Maechler M., Bolker B. & Walker S. 2015. Fitting Linear Mixed-Effects Models Using lme4. *Journal of Statistical Software* 67: 1-48.

Beck N.R., Double M.C. & Cockburn A. 2003. Microsatellite Evolution at Two Hypervariable Loci Revealed by Extensive Avian Pedigrees. *Molecular Biology Evolution* 20: 54-61.

Butler D.G., Cullis B.R., Gilmour A.R. & Gogel B.J. 2009. ASREML-R Reference Manual. Release 3.0. *Technical Report, Queensland Department of Primary Industries, Australia*

Cockburn A., Osmond H.L. & Double M.C. 2008. Swingin’ in the rain: condition dependence and sexual selection in a capricious world. *Proceedings of the Royal Society B* 275: 605-612.

Cockburn A., Osmond H.L., Mulder R.A., Green D.J. & Double M.C. 2003. Divorce, dispersal and incest avoidance in the cooperatively breeding superb fairy-wren *Malurus cyaneus*. *Journal of Animal Ecology* 72: 189-202.

Development Core Team 2011. R: a language and environment for statistical computing. Vienna (Austria): R Foundation for Statistical Computing.

Double M.C. & Cockburn A. 2003. Subordinate superb fairy-wrens (*Malurus cyaneus*) parasitize the reproductive success of attractive dominant males. *Proceedings of the Royal Society B* 270: 379-384.

Double M.C., Cockburn A., Barry S.C. & Smouse P.E. 1997a. Exclusion probabilities for single-locus paternity analysis when related males compete for matings. *Molecular Ecology* 6: 1155-1166.

Double M.C., Dawson D., Burke T. & Cockburn A. 1997b. Finding the fathers in the least faithful bird: a microsatellite-based genotyping system for the superb fairy-wren *Malurus cyaneus*. *Molecular Ecology* 6: 691-693.

Duthie A.B., Lee A.M. & Reid J.M. 2016. Inbreeding parents should invest more resources in fewer offspring. *Proceedings of the Royal Society B* 283: 20161845.

Hadfield J.D. 2010. MCMC Methods for Multi-Response Generalized Linear Mixed Models: The MCMCglmm R Package. *Journal of Statistical Software* 33: 1-22.

Jones O.R. & Wang J. 2010. COLONY: a program for parentage and sibship inference from multilocus genotype data. *Molecular Ecology Resources* 10: 551-555.

Kruuk L.E.B., Osmond H.L. & Cockburn A. 2015. Contrasting effects of climate on juvenile body size in a Southern Hemisphere passerine bird. *Global Change Biology* 21: 2929-2941.

Maguire G.S., Guay P.-J. & Mulder R.A. 2006. Isolation and characterization of microsatellite markers in the southern emu-wren (Stipiturus malachurus: Aves). *Molecular Ecology Resources* 6: 422-424.

Reid J.M. 2015. What can we really say about relatedness and extrapair paternity: a comment on Arct *et al*. *Behavioural Ecology* 26: 969-974.

Reid J.M., Arcese P., Keller L.F., Germain R.R., Duthie A.B., Losdat S., Wolak M.E. & Nietlisbach P. 2015. Quantifying inbreeding avoidance through extra-pair reproduction. *Evolution* 69: 59-74.

Vazquez A.I., Bates D.M., Rosa G.J.M., Gianola D. & Weigel K.A. 2010. Technical Note: An R package for fitting generalized linear mixed models in animal breeding. *Journal of Animal Science* 88: 497-504.

Walling C.A., Nussey D.H., Morris A., Clutton-Brock T.H., Kruuk L.E.B. & Pemberton J.M. 2011. Inbreeding depression in red deer calves. *BMC Evolutionary Biology* 11: 318-330.

Webster M.S., Tarvin K.A., Tuttle E.M. & Pruett-Jones S. 2004. Reproductive promiscuity in the splendid fairy-wren: effects of group size and auxiliary reproduction. *Behavioural Ecology* 15: 907-915.

Wickham H. 2009. *ggplot2: Elegant Graphics for Data Analysis*. New York: Springer-Verlag.

Wright S. 1922. Coefficients of inbreeding and relationship. *The American Naturalist* 56: 330-338.
